# Index of Cough Symptoms

**Published:** 1882-06

**Authors:** Geo. H. Clark, E. J. Lee

**Affiliations:** Philadelphia; Philadelphia


### INDEX OF COUGH SYMPTOMS.

ARRANGED BY GEO. H. CLARK, M. D., AND E. J. LEE, M. D.,
PHILADELPHIA.

The following index does not aim to fill the position of a treatise on cough, or diseases of the respiratory organs. Its aim is merely to bring together all the best approved symptoms relating to cough, expectoration and the respiratory organs—rendering them easy of reference.

The symptoms have been carefully and, we may add, laboriously gathered from the best authorities; and, to prevent confusion, remedies and symptoms have been numbered to correspond with authority from whom taken. It will be noticed in large blocks of remedies that there are many different numbers used. The *small* number over any remedy tells from whom that remedy was taken; the *large* number at *end* of block signifies that all remedies in that block, excepting those otherwise numbered, are from author to whom the figure refers. All symptoms, unnumbered are from Allen's Encyclopædia.

The symptoms are arranged alphabetically according to the *anatomical* part affected. Thus a cough caused by a tickling in larynx, will be found under larynx, not under tickling, or one causing soreness in stomach or head will be found under stomach or head, as the case may be, not under soreness. Many symptoms have been repeated, twice or more, to insure easy and certain reference. Some symptoms are so complex that it is a very difficult matter to decide as to how they should be placed; these have generally been arranged with reference to their most peculiar feature.

In making a prescription for any trouble of the respiratory organs, the concomitant symptoms, especially the mental, are of great importance. Yet we have been compelled to omit all concomitants that are not found in our materia medica under chest, larynx, etc. For, as any symptom of the materia medica *may* be a concomitant of cough, our task would have extended into many volumes, had we included them all. This index is intended to be an *index* to the materia medica. Before prescribing, the drug should be studied there.

It is with great pleasure, that we announce that Dr. Ad. Lippe has kindly offered to correct our MSS., each month, as it goes to press. This insures accuracy.

### Authorities Consulted.

1, Allen's Encyclopædia of Pure Materia Medica.

2, Bönninghausen's Whooping Cough.

3, Bönninghausen's Intermittent Fever.

4, Bönninghausen's Therapeutic Pocket-Book.

5, Simmon's Cough Repertory.

6, Jahr's Symptomen Codex.

7, Hering's Condensed Materia Medica.

8, Hering's Analytical Therapeutics.

9, Gross' Comparative Materia Medica.

10, Lippe's Text-book of Materia Medica (and private communications).

11, Jahr & Possart's Repertory.

12, C. Lippe's Repertory.

13, Hahnemann's Materia Medica Pura.

14, H. N. Guernsey's Lectures on Materia Medica and Notes on Cough Characteristics, *H. M.*, vol. 8, p. 322.

15, H. V. Miller, *H. M.*, vol. 8, p. 309.

16, Hull's Jahr. Repertory and Symtomatology.

17, Raue's Annual Record, six volumes.

18, N. A. J. of Homœopathy, 28 volumes.

19, R. R. Gregg's Illustrated Repertory.

20, The Cypher Repertory.

### INDEX OF SYMPTOMS

OF THE

### RESPIRATORY ORGANS.

---

### Part First—COUGH.

**ABDOMEN, aching** in left iliac region, when walking, coughing, or raising arm; nearly takes breath away; afterwards moves low down into abdomen: Eupion.

— **aching** in left rib region, on coughing and deep breathing: Val. g.

— See Bladder.

— **bruised** sensation in, on coughing: Ars.,[1] Carb. an., *Ferr*,[1] Hyos, Nux v., Plumb., Puls., Stann.[2]—10.

— **bruised** sensation in, on coughing and touching it: *Ferr.*[1]

— **bruised** sensation below ribs transversely, on coughing and on pressure, agg. by rising: Plumb.

— **burning** sensation in, with cough: Ars., Mez., Verat.—2.

— **bursting** sensation in, when coughing or eating: Anac.

— **concussion** of chest and, while coughing: Sulph.,[6] Rhus.[10]

— **concussive** pain in, when coughing: Kali c.[6]

— **constriction** of muscles of, during coughing: Lach.

— **contraction** of, on coughing: *Chelid.*, Squil.

— **contraction** of, agg. cough: Dros.[5]

**ABDOMEN, cramp** in, when coughing: Tarent.

— **cutting** pain in, with hollow cough in long shocks: VERAT.

— **deep** hollow cough, always in three or four shocks, which seem to come from the, evening: VERAT.

— **diarrhœa**, sensation in abd. as from, on coughing: *Ferr.*

— **drawing in** of, with whooping cough, so frequent can scarcely breathe; wakes at 7 A. M.: *Dros.*[7]

— **dry** cough, as if proceeding from stomach or abd., or from constipation, or as if something remained in stomach that would not pass off: SEPIA.

— **Epigastric region, bruised pain** in, from cough: *Nux v.*,[6] STANN.[1]

— — **contractive** pain in, causes cough to continue (even after sitting up): Ars.[7]

— — **emptiness** in, sensation of, with cough: Ign., Mur. ac., Stann.[2]

— — **irritation** to cough, felt in: Bar., Bry., Cham., Hepar, Lach., Nat. m., Nitr. ac., Ph. ac.—2.

— — **comes** from, cough seems to: Raph.

**ABDOMEN, Epigastric region, oppression** in, excites cough: Kali b.[2]

— — **pain** in, from cough: Ambr., Dros., Nux v.—5.

— — **small spot** in, painful to touch, violent cough appears to come from: Kali b.[6]

— — **soreness** in, from cough: Nux v.[5]

— — **stitches** in, from cough: Sep.[5]

— — **tender spot** in, seems to cause cough: Kali b.[6]

— **griping** in, and heaving as if he would vomit, from the cough, when expectoration was incomplete or difficult: Dros.[6]

— **griping** in, when coughing: Tarent.

— **Hypochondriac region, aching** in right, at night, during cough: Sulph.

— — **bruised** feeling in, and upper abdomen, on coughing: Nux v.[10]

— — **bruised** feeling in, from cough: Bry.,[5] Carbo v., Lach.—2.

— — **contractive** pain in, with cough, arresting the breathing; the cough is prevented by the pain, unless he presses with his hand on the pit of the stomach: *Dros.*[6]

— — **constriction** in, during cough: Dros.

— — **cramps** in, with cough: Zinc.[2]

— — **griping** in right, when coughing: Lycop.

— — **oppression** in, with cough: Nux v.[5]

— — **pain** in, from cough: Ambr.,[5] *Dros.*, Nitr. ac., Nux v.,[5] Oena. (r.)—1.

— — **pain** in (as if forcibly constricted), when coughing: Dros.[6]

— — **pain** in, as if ulcerated, when coughing: Lach.,[6] Puls.[10]

**ABDOMEN, Hypochondriac region, pressure** in, with cough: Acon.,[2] Ambr.,[2] Cocc., Spong., Valer—5.

— — **stitches** in, when coughing: Acon., Am. m., Ars., Bry., Lyc.,[2] Nitr. ac.,[2] Phos., Sabad., Samb.,[2] Sulph., Sulph ac.[2]—5.

— — **stitches** in right, evening, when coughing: Sepia.

— — **stitches** in left, when coughing: Bell., Carbo v., Con., Sulph., Zinc.—2.

— — **stitches** in right, when coughing: Bry., Carbo veg., Kali c., Merc., Natr. m., Sep.—2.

— — **support** it, must, with hands when coughing: Dros.[7]

— — **tearing** pain in left, when coughing: Ambr.[2]

— — **tension** in left, during cough: Hell.[7]

— — **tension** in left, causing hacking cough: Thuja.

— — **weariness** felt in, with cough: Puls.

— **Hypogastric region, bruised** feeling in, from cough: Ars., Nux v., Puls.—5.

— — **contraction** of, with cough: Dros., Squil.—5.

— — **cutting** in, from cough: Verat.[5]

— — **lancinations** in, preceding cough, as if womb would be torn off: Bell.[6]

— — **painful**, when coughing: Dros.,[7] Lyc., Nux v., Phos., Ph. ac., Sil., Squil., Verat.—5.

— — **shocks** in, when coughing: Natr. m., Squil.—5.

— — **stitches** in, with cough: Ars., Sep., Verat.—5.

— **irritation** to cough is felt in: Ambra, Ant. crud., Kali b.,[10] Sepia,[5] Thuja,[11] Verat.—2.

**ABDOMEN, lower, bruised** sensation, on coughing: Ars.[10]
— **lower, concussive pain** in, on coughing: Carbo an.[10]
— **lower, contraction** in, and sensation as if he would vomit, on coughing in evening: Dios.
— **lower, pain** in, from coughing: Sil.
— **lower, soreness,** as from coughing: Carb. veg.
— **muscles of, bruised** sensation in, on coughing: Hyos.[10]
— **muscles of, constriction** in, on coughing: Lach.
— **muscles of, contraction** in, on coughing: Squil.
— **muscles of, pain** in, on coughing: Hyos., Nux v., Squil., Sulph.
— **oppression** in, when coughing: Aur.
— **pain** (colicky), in, from coughing: Ars., *Asc. t.*, Con., *Phos.*, Phos. ac., Sulph.
— **pain** (undefined) in, from coughing: Alum., Ambra, Anac., Ars.,[12] Aur.,[11] BELL.,[12] Canth., Calc., Con., Dros., Ferr., Ipec., Kreos.,[11] Lact.,[6] Lyc., Nitrum, NUX V., Phos., *Puls.*,[11] Rhus, Sep., Sil., Squil., Stann., Sulph.,[12] Verat.—5.
— **pain in,** and chest, with coughing, in open air: Phos.[6]
— **pain** in, as if intestines would protrude on coughing: Squil.[6]
— **pain in,** and intolerance to touch, with coughing: Cham.[2]
— **pain, shooting** in, with cough: Bell., Chin., Lach., Sep., Staph.–12.
— **would protrude,** dry cough and hacking, as if its contents would: Carbo an.[6]
— **racked,** and chest and occiput, by short turns of dry cough: Lact.[6]
— **racking pain** in, with dry throat from violent dry cough: Squil.[6]
**ABDOMEN, cough seeming** to come from: Sap., Sepia.[6]
— **cough seeming** to come from, in morning: Ant. cr.
— **shaking** of, from cough: Kreos.[2]
— **shaking** of, as if everything would fall out, must hold abd. and sit, from severe, dry cough, in morning on rising: *Carb. an.*
— **shocks** in, while coughing: Puls.[6]
— **shooting** in, from coughing: *Bell.*, Chin., Lach., Sep., Staph.–12.
— **sides of,** pain in, when coughing: Con., Lycop., Squil.
— — — pain in, agg. when coughing: Bor. (r).
— **side of,** pain in, as from internal wound, when coughing, blowing nose, or putting foot down; amel. by emission of flatus: Arn. (r.)
— **side of,** stitches in, during day, from dry cough: Sulph.
— — — stitches in, on coughing: Arn., Ars.,[1] Bell. (l.), Sep.,[1] Sulph. (l.).—5.
— — — stitches in left, to small of back, when coughing: Sulph.
— — — stitches in, agg. by coughing: Carb. an. (r.), Stann. (r.)
— — — acute drawing stitches in, agg. by coughing, hiccoughing, sneezing or yawning: Bor. (r.)
— **soreness** in, from coughing: Bell.,[2] Carb. an.,[2] Con.,[2] Crot. tig., Ferr., Hyos.,[12] Nux v., Puls.[10]—5.
— **soreness** in, agg. by coughing: Sulph.
— **soreness** in, on coughing or laughing: Ars.[6]
— **soreness** in lower, as from coughing: Carb. veg.
— **stitches** in, extending, through abdominal ring, and along spermatic cord, when coughing: *Verat.*
See Stomach also.

**ABDOMEN, umbilicus**, colic as if, would be torn out, with continual cough; heat in face and sweat on forehead; after walking in open air and when lying, morning and evening: *Ipec.*[6]
— **umbilicus**, sides of, stitches in, when coughing: Sep.
— **warm**, on becoming, in, amel. cough: Sil.—5.
**ABDOMINAL RING**, aching **pain** in, when coughing: Nat. mur.
— **pain** in, from coughing: Arn.[5] *Cocc.*, Nat. mur., NUX V., Sil., Sulph., Verat.—11.
— **walls**, soreness in, and pain in stomach on coughing: Nux v.[7]
**ACHING**, see the various anatomical parts.
**ACID**, cough from nitric: Mez.[12]
— vomiting, on coughing: Nat. carb., Phos.
**ACIDS**, cough agg. by: Ant. cr., Brom., *Con.*, Lach., Nat. mur., Nux v., Sep., Sil., Sulph.—5.
**AFTERNOON**, coughing in: Agar., Alum., Amm. c., Amm. m.,[11] Ant. t., Arn., Ars., Asaf., Bad.,[12] *Bell.*,[11] Bry., Caps., Cepa,[12] Chin., Coc. c., Kali c., Laur., Lyc., Mag. c., Mez., Mosch., Mur. ac., Nat. c., Nux v., Phos., Stann., Staph., Sulph., Thuja,[12] Zinc.—5.
— — 1 to 2 P. M.: Ars.[5]
— — 3 P. M.: Coc. c.[5]
— — 4 to 8 P. M.: Lycop.
**AGITATED**, when, coughing from stitches in throat: *Cist.*
**AGED**, cough of the: Con., Hyos.
**AIR, close**, or dust, agg. dry cough at night: Nat. ars.
— **close**, agg. hacking cough: Nat. ars.
— **cold**, excites or agg. cough: Acon., *Ars.*, Aur., Bar., Bry., Bov.,[10] Carb. an., Carb. veg.,

**AIR, cold** (*Continued*).
*Caust.*, Cepa, Cham., Cina., Cist., Cupr., Hepar, Hyos., Ipec., Kali c., Kali hydrg.,[10] *Lach.*, Mez., Nux m., Nux v., *Phos.*, Phos. ac., Rhus, Rumex, Samb., Sep., Sil., Spon., Stram., Sulph.—2.
— **cold**, amel. cough: Coccion.
— **icy cold**, seems to stream through air-passages on deep inspiration, with desire to cough: *Coral. r.*
— **cold,** persistent coughing after walking in, also when lying down, excited by deep inspiration, accompanied by colic, as if umbilicus would be torn out, heat in face, and sweat on forehead: Ipec.[6]
— **damp cold**, agg. cough: Ant. t., Calc., Carb. an., Carb. veg., Chin., Dulc., Lach., Mag. c., Mosch., Mur. ac., Nit. ac., Sulph., Sul. ac., Verat., Zinc.—2.
— **draft** of, agg. cough: Acon., Caust., Chin.—2.
— **dry cold**, agg. cough: Acon., Cham.,[2] Samb.,[2] Brom. Phosph., Hepar., Nux m., Spong.—10.
— **entering cold**, agg. cough: *Carb. veg.*, Phos.
— **gasping for**, before each paroxysm of cough: Ant. t., Bry., Coc. c., Coral. r.
— **inspiring cold**, agg. cough: *Cepa*, Cist., Cupr., Rumex,[2] Staph.,[2] Vit.—5.
— **inspiring cold**, causes hacking cough: CEPA, Phosph.[10]
See also Inspiration and Expiration.
— **walking in cold**, agg. cough: Ars., Ipec. Phosph.[10] —I.
— **warm room**, going from, to cold air, or *vice versa*, causes coughing: Sepia., Nux v.,[10] Natr. carb.[10]
— **night**, agg. cough: Merc.

**AIR, open**, excites or agg. cough: Acon.,[12] Alum.,[11] ARS , Bar., Bry., Calc., Carb. veg., Cham., Cina, Cocc., Dig., Ferr.,[12] Ipec., Kali b., *Lach.*,[11] Lyc., Mosch., Nit. ac., NITRUM., Nux v., *Phos.*, Phos. ac.,[12] Rhus, Rumex, Seneg., Sil., Spig., Staph., Stram.,[12] Sulph.,[12] Sul. ac. —5.

— **open**, coughing in, with pain in abdomen and chest: Phos.[6]

— — at night, causes cough: Calc. p., Linu., Phos., Spig., Sulph., Sul. ac., Trif. p.

— — amel. cough: Nux v.[5]

— — amel. dry cough: Iod,.[1] Nux v.[5]

— — causes dry cough: Spig.

— — entering, agg. cough: Ipec., Bry., Rumex, Squil.—12.

— — agg. hacking cough: Seneg., Sulph.

— — amel. hacking cough: Lil. tig.

— — amel. rough cough: Iod.

— — short cough from: Spig.

— — agg. tickling cough: *Lach.*

— — going into warm room from, excites cough: Acon.,[12] Ant. cr.,[7] Bov.,[1] Coc. c.,[10] Natr. carb.,[10] Verat.[10]

— — on entering warm room from cold, feels sensation in trachea as if full of smoke, which excites cough; feels as if he could not inhale sufficient air: Bry.[6]

— — going from warm room to, excites cough: *Acon.*

**AIR PASSAGES, burning** in, with cough: Ant. cr., Carb. veg., Caust., Cina, Iod., Lach., Mag. m., *Spong.*, Sulph., Zinc.—11.

— **catarrh of**, dry cough at night in bed, as if from: Coca.

— **crawling** in, at night, causes hacking cough: Æth.

— **crawling** irritation near suprasternal fossa, before midnight, causes cough, agg. by swallowing mucus: Apis.

**AIR PASSAGES, dryness** of, causing cough: Carb. an., Lach., Merc., Petrol., Puls.—11.

— **dull cutting**, from below upwards in, which becomes a stitch and excites two or three fits of coughing: *Arg.*

— **irritation** in, causing coughing: Ars., Colch., Kali b.

— **irritation** in, causing hacking cough at night: Kali b.

— **irritation** in, to cough: Chlo.

— **irritation** in, when coughing: All. s.

— **irritation** in, in evening, causes cough: Sulph.

— **irritation** in, in evening, in bed, causes cough: Agnus, Amm. c., Coff., Kali c.

— **irritation** in, low down, causing coughing at night: Cham.

— **tickling irritation** in, causing cough: Ars., Calc. ac.,[1] HYOS.,[1] Kali b., Nux. v., Puls., Staph., Verat.—10.

— **tickling irritation** in, as if it would provoke coughing, makes breath short, and is amel. by moderate exertion: *Rhus.*

— **tickling irritation** in small spot: Apis, *Con.*—10.

— **irritation** in, upper part, causing coughing: Plan.

— **mucus** gets into, on stooping, or going up-stairs, which is expelled by a single cough: *Arg.*

— **pain** in, agg. when coughing: Camph.

— **raw**, pain as if, with coughing: Ambr., Anac., Ant. cr., *Arg.*, Arn., Calc., Carb. veg., Grat., Kali c., Kreos., Laur., Mag. c., Mez., Nux mos., Petrol., *Phos.*, Ruta, Sep , Sil.—11.

— **rawness** of, causes coughing: *Coc. c.*, Stann.

**AIR PASSAGES**, **rawness** of, causes coughing, 10 A. M., while lying: Coc. c.
— **sore**, pain as if, with coughing: Alum., Ambr., Amm. c., Arg., Ars., Baryt., *Bell.*, Calc., CARB. VEG., *Caust.*, Chin., Cina, Hep., Lach., Lycop., Mag. m., Merc., Nat. c., Nux mos., Nux vom., PHOS., Sep., Sil., Spig., Spong., STANN., SULPH.—11.
— **stitches** in, with cough: Kali c., Lach., Phosp.—10.
— **stitches** in, extending upward, causing coughing: *Arg.*
— **tickling** in, causing coughing: ACON., Amm. c., Amm. m., Angus., Ant. t., Arg. n., *Arn.*, Asaf., Bar., Bell., Bor., Bov., *Brom.*, Bry., Caps., *Carb. an.*, *Carb. veg.*, *Caust.*, CHAM., Chin., Cina, Coc. c.,[1] Colch., *Con.*, Cupr., Dig., Ferr., Graph., Hepar, Ign., IOD., IPEC., *Kali b.*, Kali c., LACH., Lact., Laur., Led., Mag. c., Mag. m., Marum, Merc., Mur. ac., Nat. c., NAT. M., Nitrum, NUX VOM., Olean., *Phos.*, Prun., *Puls.*, Rumex,[10] Sabin., Sant.,[1] Seneg., SEP., Sil., *Spon.*, *Stann.*, STAPH., Sulph., Verat., Zinc.—11.
— **tickling** in, at night, causing coughing: Rumex,[10] Sant.[1]
— **tickling irritation** in, causing coughing: Calc. ac., *Hyos.*
— **tickling** low down in, causing coughing: Cina.
— **wall**, **posterior**, of, **tickling** low down in, causing coughing, agg. by lying and sleeping, amel. by loosening mucus: Apis.

**AIR**, warm, agg. coughing: Ant. cr., Cocc. c.,[10] Iod.—12.

**ALTERNATING with coryza**, cough: Colch.
— with headache, cough: Lach.

**ANGER**, coughing **from**: Acon., Ant. t., Ars., *Cham.*, Chin., *Ign.*, Nux v., Sep., *Staph.*, Verat.—2.

— **before** coughing: Asarum.

— **from the coughing**: Acon.[5]

— with **fright**, cough from: Acon., Ign.—2.

See Vexation.

**ANGRY**, **when getting**, violent spell of coughing comes on: Ant. t., Arg. nit.,[7] Arn., Asar., Cham. —8.
— coughing if child gets, vomits food and mucus: Ant. t.[2]

**ANGUISH**, accompanying coughing: *Acon.*, Cina, *Coff.*, *Dig.*, Hepar., Iod., Rhus.—11.
See Vexation.

**ANUS**, pain in, with coughing: *Lach.*[6]

**ANXIOUS**, cough: Ars., Cina, Coff., Rhus.—5.
— cough **before midnight**, on waking: *Rhus.*

**ANXIOUSNESS**, **obstruction** of breath, pale face and whimpering, cough ending with: Cina.

**ANXIETY**, **with coughing**: *Acon.*, Cina, *Coff.*, Dros., Eupat., *Hepar.*, Iod.,[12] Rhus., Samb., Spon., Stram.—5.
— **nocturnal**, with coughing: *Acon.*[12]

**APHONIA**, cough with: Amm. caust.,[10] Phos.,[12] Spong.[10]

**APPETITE**, **cough**, with loss of: Podoph.

**APPREHENSION**, **discouragement** and, following short cough, caused by severe tickling and irritation behind upper sternum: RHUS.[1]

**ARMS**, **on becoming cold**, cough from: Ars., Calc., Ferr., Hepar.,[5] Kali c., Rhus.,[7] Sil.[2]—12.

